# Impact of Moisture-Buffering Materials and Time-Averaging of Infiltration Rate on Prediction of Indoor Air Humidity

**DOI:** 10.3390/ma19214475

**Published:** 2026-10-23

**Authors:** Dobrosława Kaczorek, Małgorzata Basińska, Tomasz Kisilewicz, Katarzyna Nowak

**Affiliations:** 1 Thermal Physics, Acoustics and Environment Department, Building Research Institute (ITB), Filtrowa 1 Str., 00-611 Warsaw, Poland; d.kaczorek@itb.pl; 2 Faculty of Environmental Engineering and Energy, Institute of Environmental Engineering and Building Installations, Poznan University of Technology, Berdychowo 4 Str., 61-131 Poznan, Poland; malgorzata.basinska@put.poznan.pl; 3 Department of Building Design and Building Physics, Faculty of Civil Engineering and Transport, Cracow University of Technology, Warszawska 24 Str., 31-155 Krakow, Poland; knowak@pk.edu.pl

**Keywords:** moisture buffering, air infiltration, hygrothermal simulation, indoor relative humidity, infiltration temporal averaging

## Abstract

**Highlights:**

- Moisture buffering, combined with efficient air exchange, can balance high moisture emissions.
- Highly variable air infiltration is an essential factor in internal humidity comfort.
- The unpainted clay board proved to be a very efficient moisture buffer.
- A simplified infiltration model may lead to false interpretations of high moisture emissions.

**Abstract:**

The moisture buffering properties of materials and actual air exchange are crucial for indoor conditions in buildings. Consequently, precise material data and an adequate model of highly variable outdoor air infiltration are essential for computer-based design simulations. This study quantifies how internal cladding materials and infiltration time-averaging affect indoor relative humidity prediction. Three internal finishing variants were analysed: painted gypsum plasterboard, painted clay board, and unpainted clay board, the latter characterised experimentally by a practical moisture buffer value of 1.88 g/(m^2^·%RH). Three moisture-load scenarios were considered, resulting in 27 simulation cases. Hourly infiltration rates were calculated in the DesignBuilder software and then converted into annual, monthly, and daily averages for WUFI Plus 3.X simulations. The annual mean infiltration rate was 0.117 h^−1^, while selected daily means ranged from 0.03 to 0.60 h^−1^. Passive moisture buffering, especially the unpainted clay board, reduced short-term RH fluctuations but did not entirely compensate for insufficient moisture removal. Surface finishing has a decisive influence on the practical performance of hygroscopic materials. Under high moisture gains, median RH reached about 69.5%, and predictions became sensitive to infiltration resolution. Annual averaging may therefore be sufficient for low moisture loads, whereas daily values are required when short-term moisture peaks and moisture buffering effects are relevant.

## 1. Introduction

Accurate prediction of indoor humidity and temperature conditions is crucial in building performance simulation, especially for energy-efficient structures where factors like air tightness, heat loss from ventilation, and indoor environmental quality must be addressed simultaneously [1,2,3]. In such buildings, mechanical ventilation is typically designed to ensure adequate hygienic air exchange, while uncontrolled infiltration is expected to be limited. However, even in relatively airtight envelopes, infiltration remains a variable and unpredictable aspect of the overall air exchange rate. Its magnitude depends on outdoor temperature, wind speed and direction, pressure differences across the envelope and building airtightness [4,5]. Consequently, infiltration is not a steady boundary condition but rather a dynamic process that can significantly modify indoor humidity levels.

Despite this variability, air infiltration is frequently represented in hygrothermal and building performance models by constant or averaged values over time, or is entirely overlooked [6,7,8,9,10,11]. Such simplification is practical and computationally convenient, but it might not align with the actual physical characteristics of the process. This is particularly important for predicting indoor relative humidity because humidity responds not only to mean air exchange but also to short-term interactions between ventilation, infiltration, moisture gains and moisture storage in internal surfaces [4,12,13,14]. Consequently, the chosen temporal resolution for infiltration modelling can affect the estimated number of hours within an acceptable humidity range and the evaluation of moisture-related performance. 

Indoor relative humidity is important for both occupant comfort and building durability [15]. Excessively low RH may reduce perceived comfort, whereas high RH may support surface condensation, material degradation and mould growth if maintained for sufficiently long periods [12,15]. In residential buildings, indoor RH is shaped by moisture emissions from occupants and household activities, operation of ventilation systems, outdoor climate, and the hygroscopic properties of internal finishing materials [16,17,18,19]. In energy-efficient buildings, this balance is especially sensitive because reduced uncontrolled air exchange may limit moisture removal, while high airtightness increases the importance of correctly representing the remaining infiltration component.

Materials that buffer moisture are able to dampen brief changes in humidity by taking in water vapour when relative humidity is high and discharging it as the surrounding air dries out. Research has confirmed this phenomenon in various porous finishes, including clay and wood products, cement-based composites, and plasters [6,12,20,21,22,23,24,25,26]. The moisture buffer value (MBV), often measured via the NORDTEST protocol, serves as a practical metric for assessing this material property [6]. Nevertheless, the overall performance of moisture buffering within a room is not solely reliant on the MBV; it is also influenced by the interplay of hygroscopic surfaces, the patterns of moisture loads, and air exchange factors like ventilation and infiltration [5,13,27]. Even materials with strong buffering capabilities might see their impact on humidity peaks restricted by finishes, thin layers, or constant exposure to high moisture [13,28].

Previous research has explored how ventilation and infiltration impact indoor moisture levels, alongside several investigations into the effects of hygroscopic materials on relative humidity [4,12,13,14,29,30,31]. Nevertheless, the effect of simplifying the time scales in infiltration models has not been widely studied. Many simulations treat infiltration as a constant annual value, a monthly mean, or omit it altogether [6,7,8,9,10,11]. This creates a methodological question regarding when a mean infiltration rate is adequate for evaluating indoor humidity versus when more detailed temporal data is necessary. This issue is crucial for evaluating moisture-buffering materials in modern, energy-efficient buildings with mechanical ventilation, as simplistic infiltration models might inaccurately represent the materials’ effectiveness in stabilising humidity.

This research addresses this gap by measuring how time-based infiltration averages affect the forecasting of indoor relative humidity within a typical home environment. Initial infiltration data was obtained on an hourly basis through DesignBuilder [32], which was subsequently transformed into yearly, monthly, and daily averages for use in hygrothermal modelling with WUFI Plus [33]. Three types of interior finishes were examined: gypsum plasterboard with paint, clay board with paint, and raw clay board. Through standardised lab tests [34,35], the hygrothermal characteristics and moisture-buffering capabilities of the clay board were empirically determined. The study utilised three distinct moisture-load profiles to reflect low, standard, and high levels of household moisture production [16,17,18,19].

Although considerable attention is nowadays devoted to the precise modelling and thermal assessment of the indoor environment, a somewhat different approach is taken regarding humidity-related aspects and the factors influencing them. The authors aim to demonstrate the extent to which the combination of finishing materials and uncontrolled infiltration-driven air exchange can affect (negatively or positively) these conditions, especially in cases of high moisture loads.

The objective of the study is to investigate the influence of temporal infiltration modelling on projected indoor relative humidity and the characterisation of moisture-buffering materials. Focused on the 40–60% RH comfort interval, the study contrasts annual, monthly, and daily infiltration data within uniform boundary settings, ultimately advising on the best modelling strategy based on hygroscopicity, moisture levels, and the necessary degree of hygrothermal precision.

The strong dependence of the indoor microclimate on external conditions makes it impossible to directly apply the results and observations to other locations. However, the authors hope that the results presented here will encourage similar studies in different climatic conditions.

## 2. Materials and Methods

### 2.1. Approach to the Research

This section presents the results of fundamental tests on the thermal and moisture-related properties of prefabricated clay board, which may serve as a favourable alternative to gypsum plasterboard, commonly used as an internal finishing material. The section also outlines the adopted approaches for modelling infiltration airflows and moisture loads in the simulations. Hygrothermal simulations were conducted for three variants of internal cladding material. The first variant used the experimentally tested, prefabricated painted clay board (A) as the wall surface material. The second variant employed the same clay board without surface painting (A*), and the third variant, serving as the baseline, consisted of painted gypsum plasterboard (B). By analysing the resulting humidity conditions within the room, the study assessed how the simplifications adopted in the model for infiltration airflow influence the obtained results of hygrothermal simulations. The analyses focused on relative humidity (RH) values, as RH is a component of thermal comfort parameters and is closely linked to moisture buffering capacity (MBC).

### 2.2. Laboratory Measurement and Data Preparation

The laboratory tests, conducted at the laboratories of the Department of Building Physics at Cracow University of Technology and at the Building Research Institute (ITB) in Warsaw, focused on determining the hygrothermal properties of a clay board that was new to the Polish market. 

#### 2.2.1. Thermal Conductivity

The thermal conductivity coefficient of the clay board (A*) was measured using the FOX 314 apparatus, manufactured by TA Instruments, New Castle, DE, USA, with a measurement range of 0.01÷0.20 W/(m·K) and an accuracy of 1%. The measurements were conducted under steady-state heat flow conditions in accordance with the standards: PN-EN ISO 8301:1998 [36], PN-EN 12667:2002 [37], and ASTM C518-91 [38].

The study was conducted on three samples, each with dimensions of 305 × 305 mm and a thickness of 16 mm. Prior to testing, the samples were conditioned at temperatures of (23 ± 5) °C and a relative air humidity of (50 ± 5)% until a constant mass was achieved. Measurements were carried out at an average sample temperature of 10 °C, with a temperature difference across the thickness of the sample of 20 K and heat flow directed from bottom to top.

#### 2.2.2. Moisture Buffering Value

The practical moisture buffering value (MBV_practical_) was determined as the amount of moisture transported through a unit exposed surface area of the material, relative to a unit change in relative humidity (ΔRH). The result is expressed in units of g/(m^2^·%RH) (Equation (1)).

(1) $${\mathrm{MBV}}_{\mathrm{Practical}}=\frac{∆m}{S\cdot \left({\mathrm{RH}}_{\mathrm{high}}-{\mathrm{RH}}_{\mathrm{low}}\right)}$$

where: Δ*m* is the mass gain/loss during a given cycle, in grams (g); *S* is the exposed surface area of the sample, in square metres (m^2^); *RH_high/low_* is the high or low level of relative humidity, expressed as a percentage (%).

The measurement of MBV_practical_ was carried out in accordance with the procedure of the NORDTEST method [6], in which the material samples were subjected to step changes in relative humidity over a 24 h cycle: 75% RH for 8 h and 33% RH for 16 h. For each cycle, two values of mass change were calculated: mass gain during adsorption (*m*_8h_ *− m*_0_) and mass loss during desorption (*m*_24h_ *− m*_8h_). The average of these two values was then determined. The final value of *MBV_practical_* was calculated as the mean from at least three stable cycles. 

The ideal moisture buffering value (*MBV_ideal_*) was calculated for comparison with the experimentally determined *MBV_practical_* value. The *MBV_ideal_* is a parameter calculated at the material level and is derived from an analytical equation based on Fick’s principle regarding the diffusion of water vapour in a porous material. *MBV_ideal_* can be calculated using Equation (2) [20]:

(2) $${\mathrm{MBV}}_{\mathrm{ideal}}=0.00568\cdot p_{\mathrm{sat}}\cdot b_{m}\cdot \sqrt{t_{p}}$$

where *p_sat_* is the water vapour saturation pressure at 23 °C, Pa; *b_m_* is the moisture effusivity, kg/(m^2^⋅Pa⋅s^1/2^); and *t_p_* is the time period used during the test, s.

To determine moisture effusivity (*b_m_*), Equation (3), provided by Rode et al. [39], was used:

(3) $$b_{m}=\sqrt{\frac{\delta_{p}\cdot \xi_{w}}{p_{\mathrm{sat}}}}$$

where *δ_p_* is the water vapour permeability, kg/(m s⋅Pa), and *ξ_w_* is the moisture capacity, kg/m^3^.

The moisture capacity is usually determined by the slope of the adsorption curve, given by Equation (4):

(4) $$\xi_{w}=\frac{\partial_{w}}{\partial_{\varphi }}$$

where *w* is the equilibrium moisture content, kg/m^3^, and *ϕ* is the RH.

The moisture capacity (*ξ_w_*) is considered as average values within the relative humidity range of 33–75%, as these are the humidity levels accounted for in the NORDTEST method [6]. The derivation of *MBV_ideal_* is based on three assumptions: (1) the material is homogeneous; (2) the material thickness exceeds the moisture penetration depth; (3) the relative humidity variation occurs at the material surface, meaning the surface vapour transfer coefficient at the boundary is assumed to be infinite.

Furthermore, the moisture penetration depth (*d_p_*) is defined as the distance between the material surface and the depth where the amplitude of the vapour pressure variation is 1% of that on the surface [40,41]. It is given by Equation (5):

(5) $$d_{p,1\%}=4.61\sqrt{\frac{D_{w}\cdot t_{p}}{\pi }}$$

where *D_w_* is the moisture diffusivity of the material, m^2^/s, calculated as:

(6) $$D_{w}=\frac{\Delta_{a}\cdot p_{\mathrm{sat}}}{\xi_{w}\cdot \mu }$$

where *δ_a_* is the vapour permeability of air, kg/(m·s·Pa); *µ* is the vapour resistance factor of the material; and *t_p_* = 24 h is the load cycling period.

#### 2.2.3. Water Vapour Resistance Factor

To determine the water vapour resistance factor, the dry cup method was employed according to the ISO 12572 standard [34]. Prepared samples were initially conditioned at 23 °C and 50% RH in the climatic chamber until they reached a constant mass.

Then, the samples were placed in special metal cups containing a desiccant (CaCl_2_), manufactured by Biomus, Poland. The test samples were sealed with wax into the cups to prevent moisture infiltration except for the upper surface of the samples. Each cup was then weighed every 24 h until a quasi-steady state was reached, when the difference between two consecutive weighings became constant within 5%. The water vapour resistance factor *µ* was calculated according to Equation (7):

(7) $$\mu =\frac{\delta_{a}\cdot S\cdot ∆p}{G\cdot d}$$

where *G* is the water vapour flow rate, kg/s; *d* is the test sample thickness, m; *S* is the exposed surface area, m^2^; and Δ*p* is the water vapour pressure difference across the sample, Pa.

Since the vapour resistance factor of clay-based materials may depend on relative humidity, an additional sensitivity analysis was performed to test the robustness of the constant *μ* assumption adopted in the simulations. The measured dry cup value for the analysed clay board (*μ_dry_* = 17.4) was used as the baseline case. Additional simulations were carried out for *μ* = 10, 8 and 6, representing a literature-supported range of more vapour-open behaviour at higher humidity levels. This range is consistent with published data for clay boards, reported for a CLAYTEC clay board datasheet [42] (*μ* = 5–10) and the humidity-dependent values reported by Goto et al. [43] for clay board, decreasing from 8.7 at 25% RH to 6.1 at 86.5% RH. The selected range is also in line with Hall et al. [44], who showed that the vapour resistance factor generally decreases with increasing mean relative humidity and that wet cup values are consistently lower than dry cup values for a given material.

#### 2.2.4. Sorption Isotherm

A sorption isotherm test in a climate chamber was conducted in accordance with the PN-EN ISO 12571:2022 standard [35] on three samples of prefabricated clay board (A*) with a density of 1500 kg/m^3^, dimensions of 200 × 120 mm, and a thickness of 16 mm. In this test, a drying temperature of 60 °C was adopted, which ensured effective moisture removal while maintaining the integrity of the tested samples (due to the presence of organic additives in the board—straw). Each sample was dried until a constant mass was reached (defined as a mass change of no more than 0.1% of the total mass over a 24 h interval). To determine the adsorption isotherm, the mass of the sample was measured until its constant mass was reached at each stage of gradually increasing relative humidity levels—20, 30, 40, 50, 60, 70, 75, 80, 85, 90, and 95%—at a constant temperature of 23 °C ± 0.5 °C [35] (temperate climate conditions).

### 2.3. Building Simulation Model

Room-scale hygrothermal simulations were performed in WUFI Plus [33]. This software has been verified in the IEA Annex 41 project [45] and validated experimentally in previous studies [46]. In the present work, WUFI Plus was used for comparative scenario analysis; therefore, the emphasis was placed on maintaining identical boundary conditions across variants rather than on calibrating a monitored case study.

It was assumed that during the heating season, from 1 October to 30 April, the reference indoor temperature of 20 °C is maintained by a condensing gas boiler (with a minimum efficiency of 70% [47]). During the cooling season, from 1 June to 31 August, the reference indoor temperature of 26 °C is maintained by an air conditioner (with an average coefficient of performance (COP) of 3). Due to the climatic conditions in Poland, a transitional period was assumed (from 1 May to 31 May), during which neither the heating nor cooling systems were in operation.

#### 2.3.1. Room Characteristics

In the software, a hypothetical model of a room located on the top floor of a multi-family residential building was defined. The height of the three-story building is 9 m above ground level. It was assumed that the analysed room, with a volume of 59 m^3^, serves as a family living room connected to an open-plan kitchen area. The room is a corner unit with two external walls. The wall with a window opening of 2.9 m^2^ faces south, while the other external wall faces west. The intermediate floor slab and internal walls were treated as adiabatic partitions. The thermal properties of individual building components (Table 1), except the roof, were adopted in accordance with the thermal protection requirements for buildings in force in Poland in the year 2025 [47]. The baseline variant (B) assumed internal finishing of the building partitions with painted gypsum plasterboard. As additional variants, the use of prefabricated clay boards was analysed—both painted (A) and unpainted (A*)—as replacements for the gypsum plasterboard. These modifications did not noticeably affect the thermal transmittance (*U*-value) of the building partitions and correspond to the actual commercial thicknesses of these materials. The physical properties of the prefabricated clay board (A*) were determined on the basis of laboratory tests, and their detailed description is provided in Section 3.

#### 2.3.2. Weather Data

The simulations were performed for Kraków, Poland (50.0647° N, 19.9450° E), using the typical meteorological year for this location [48]. Kraków has a temperate climate with cold winters and warm summers; therefore, the selected climate provides seasonal variation in both temperature-driven and wind-driven infiltration. Wind-driven rain (WDR) was not included as an external moisture load. Although WDR can be relevant for envelope hygrothermal response [49], the present study focuses on indoor humidity driven by ventilation, infiltration and internal moisture gains. This simplification was considered acceptable for the analysed externally insulated multilayer wall, where the influence of WDR on the internal surface moisture balance is expected to be limited. Previous studies have shown substantial WDR effects mainly for non-insulated historic masonry or internally insulated brickwork [50,51,52,53], which differs from the envelope configuration analysed here. Accordingly, WDR was excluded consistently in all variants to maintain a controlled comparison of infiltration-averaging assumptions.

#### 2.3.3. Internal Gains and Schedules

In the analysed case, it was assumed that the room represents a daytime living area in an apartment—a kitchen combined with a living and dining room—used by two adults and one child. The room is used by residents between 6:00 and 23:00. At night, residents stay in another room that serves as a bedroom. For the purposes of the simulation, various scenarios of room usage were adopted depending on the day of the week, with separate assumptions for weekdays and weekends, taking into account the varying activity patterns of the household members.

In order to assess the relative humidity levels within the residential room, it was essential to account for a moisture-load scenario based on typical sources of water vapour emission.

In this study, three composite indoor moisture-load scenarios, commonly applied in the literature [16,17,18,19], were adopted: low (L), typical (T), and high (H). These scenarios were defined by different combinations of moisture emissions from occupants and household activities rather than by proportional scaling of each individual source. Consequently, the values assigned to a particular activity do not necessarily increase consistently from L to H. Table 2 presents the unit moisture emissions adopted for individual household activities, including meal preparation [19].

The unit emission values presented in Table 2 were combined with the adopted occupancy and activity schedules to generate the total hourly moisture-load profiles presented in Table 3.

#### 2.3.4. Assumptions Regarding Infiltration and Ventilation Rate

It was assumed that the simulated facility is equipped with a mechanical supply and exhaust ventilation system (S-EX) that, regardless of external conditions, ensures the required air exchange in the room. The supply airflow rate was assumed to be one exchange per hour, i.e., 60 m^3^/h, based on the number of occupants in the room (3 people) and the unit volume of supply air according to Polish technical regulations at 20 m^3^/(h·person) [47]. The operation of the mechanical ventilation system corresponds to the room usage scenario presented in Table 2. In the analyses, air infiltration, being a function of the assumed building airtightness level of *n*_50_ = 1.5 h^−1^ and the external conditions surrounding the building, was determined using the DesignBuilder software, version DB7.0.2.006 [32]. DesignBuilder is a convenient interface to the widely used around the world Energy Plus software. Calculation of infiltration was based on the design flow rate model. In this model, the user defines a design flow rate that is modified by the momentary temperature differences and wind speed. The hourly values obtained for the entire year were then utilised in moisture analyses conducted with the WUFI Plus software, where they were averaged to derive annual (a), monthly (m), and daily (d) mean values.

### 2.4. Variants—Abbreviations

The study presents variant analyses for:

- Three types of finishing materials:
  ○ Painted gypsum plasterboard (base variant)—B;
  ○ Painted clay board—A;
  ○ Unpainted clay board—A*.
- Three levels of moisture gains in the room:
  ○ Low—L;
  ○ Typical—T;
  ○ High—H.
- Three variants of simplified air infiltration modelling:
  ○ Hourly values averaged to a constant annual value—a;
  ○ Hourly values averaged to a constant monthly value—m;
  ○ Hourly values averaged to a constant daily value—d.

### 2.5. Evaluation Criteria and Study Framework

The results from the simulations (hourly values) allowed for the assessment of the variability of relative humidity in the room depending on the level of simplification in the approach to air infiltration, the type of finishing material used, and the moisture gains. To evaluate the impact of the simplification, a criterion based on the variability in the number of hours during which the indoor relative humidity remained within the acceptable range, defined as 40–60%, was applied. Additionally, the authors’ own evaluation method was proposed using percentage-hour indicators, *Ph* [%h], as described by Equation (8), giving RH values that fell outside the acceptable range.

(8) $$\mathrm{Ph}=\left\{\begin{array}{c} \sum_{i=1}^{h}{\left({\mathrm{RH}}_{\mathrm{in}}-60\right)}_{i}\;\mathrm{for}\;{\mathrm{RH}}_{\mathrm{indoor}}>60\%\\ \sum_{i=1}^{h}{\left({40-\mathrm{RH}}_{\mathrm{in}}\right)}_{i}\;\mathrm{for}\;{\mathrm{RH}}_{\mathrm{indoor}}<40\% \end{array}\right.$$

where: *h*—number of hours, *RH_in_*—relative humidity of indoor air, %.

The analysed construction and installation solutions were evaluated using the following statistical measures: the median (central value); the range (difference between the highest and lowest observed value); and the coefficient of variation (CV), i.e., the percentage of the mean represented by the standard deviation, as described by Equation (9).

(9) $$\mathrm{CV}=\frac{\mathrm{SD}}{x}\cdot 100$$

where: *SD*—standard deviation, %RH; *x*—arithmetic mean, %RH

**Figure 1:**
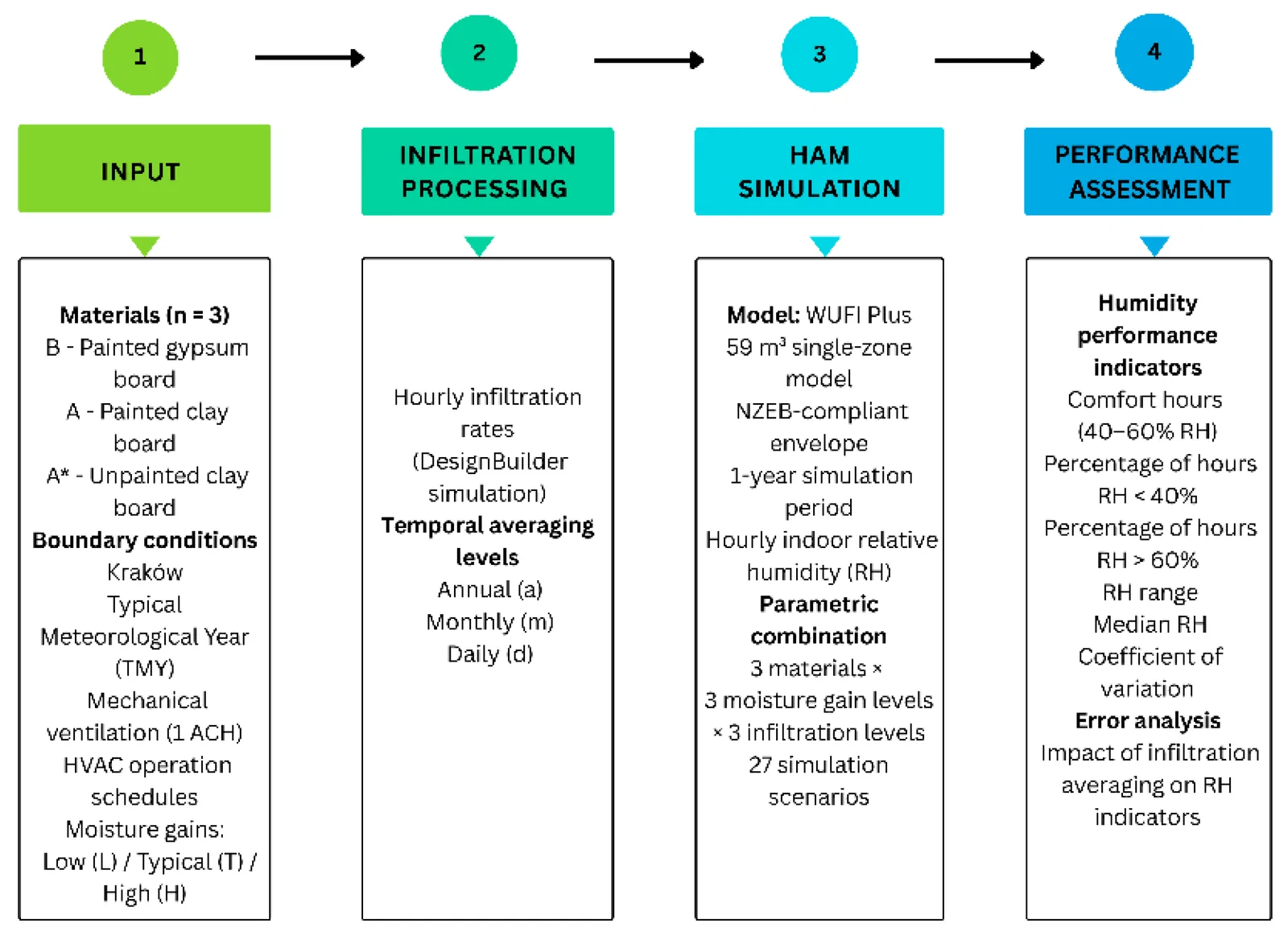
Study framework summarising input parameters, infiltration processing, HAM simulation structure and humidity performance assessment.

The modelling components and evaluation criteria described above constitute a closed and internally consistent study framework. The complete structure of the study, integrating material variants, boundary conditions, hourly infiltration modelling and temporal averaging procedures within the conducted simulations, is presented in Figure 1. Mechanical ventilation and all other boundary conditions remained unchanged across individual simulations. This approach led to the development of 27 distinct simulation scenarios, which formed the basis for subsequent analyses.

## 3. Laboratory Test Results and Data Preparation Results

The aim of the laboratory tests conducted was to obtain the necessary data concerning the hygrothermal properties of the analysed interior surface finishing materials used in the simulation analyses. 

### 3.1. Hygrothermal Properties Determined in Laboratory Tests

The laboratory tests provided the material parameters required to define the internal finishing layers in the hygrothermal simulations. Table 4 presents the equilibrium moisture content as a function of relative humidity for the analysed unpainted clay board (A*). For comparison, the corresponding data for gypsum plasterboard (B), taken from the WUFI Plus database, are also included.

The laboratory tests also enabled the determination and calculation of the fundamental hygrothermal properties of the unpainted clay board (A*)—Table 5. The measurements were performed with an expanded uncertainty of *U*(RH) = 0.058% RH for relative humidity and *U*(w) = 0.017 kg/m^3^ for moisture content.

The measurements of *MBV_practical_* were carried out with an expanded measurement uncertainty of *U*(MBV) = 0.05 g/(m^2^·%RH). The *MBV_practical_* value obtained from the measurements is higher than *MBV_ideal_*, which contradicts the findings reported by Larcher et al. [54], where the opposite relationship was observed. However, it should be noted that the measured and ideal *MBVs* are comparable only when the material is homogeneous, and the thickness of the tested sample is equal to or greater than the depth of moisture penetration [2]. In the conducted experiment, the calculated moisture penetration depth for the clay board (A*) was approximately 2.5 cm, which significantly exceeds the thickness of the tested sample (16 mm). Similarly, in the study by Wang and Lu [55], the *MBV_practical_* value was higher than *MBV_ideal_*, which was explained by the nonlinear nature of moisture diffusion in reality.

### 3.2. Hygrothermal Properties from the WUFI Database

Additional data necessary for conducting simulation analyses in the WUFI Plus software for the analysed finishing materials were obtained from the programme’s database (Table 6).

In the simulations, both the gypsum plasterboard (B) and clay board (A) were assumed to have an acrylic resin-based paint finish with the same equivalent air layer thickness of *s_d_* = 0.5 m [28]. In WUFI Plus, the coating was represented as an additional vapour diffusion resistance at the room-facing surface, without introducing a separate paint layer into the assembly. The *s_d_* value therefore describes the overall vapour diffusion resistance of the coating system, rather than its physical dry-film thickness. Coating thickness and the number of application coats were not specified independently. The unpainted clay board (A*) had no additional coating resistance.

## 4. Results

### 4.1. Infiltration and Ventilation Rate

**Figure 2:**
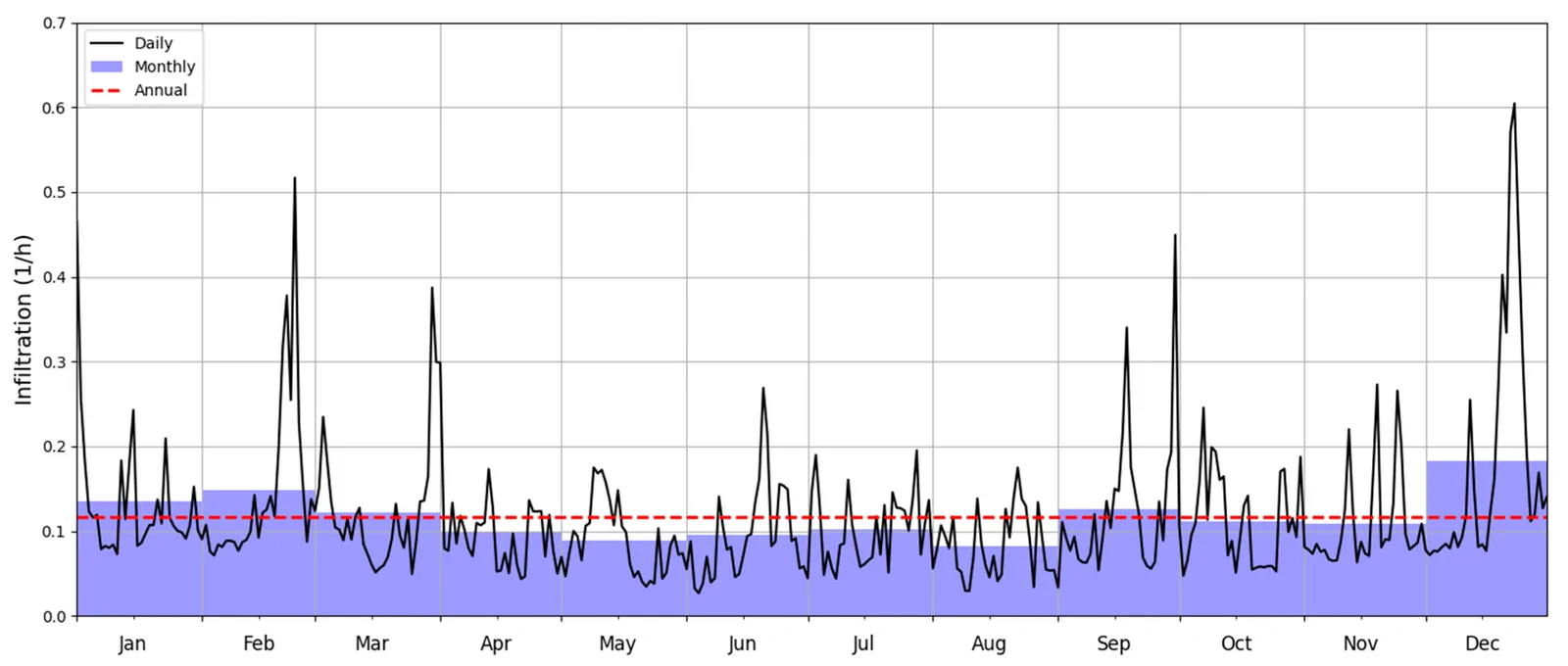
Variability of air infiltration, monthly and annual average values.

In order to assess how the adopted simplifications in infiltration modelling affect indoor relative humidity (RH) levels, the variability of the simulated infiltration rates was analysed by determining average values for the examined scenarios. While analysing the variability of indoor air infiltration, a very high variability in hourly infiltration values throughout the year was observed. The monthly infiltration level is highest in December and the lowest in August. The maximum value was recorded in December at 0.769 L/h, and the minimum in August at 0.001 L/h. Assuming that the supply and exhaust ventilation system in the building ensures an airflow of 1.0 L/h, the share of infiltration in the total air exchange is 43% for the maximum level and only 0.1% for the minimum value. For the hourly data across the entire measurement period, the data range is 0.58 h^−1^, while the data ranges for August and December are 0.45 and 0.71 h^−1^, respectively. This indicates that December is characterised by a significant variability in infiltration levels, which may have a considerable impact on indoor conditions. To show this variability, Figure 2 shows the average monthly values (m), the average daily (d) values in comparison to the average annual value (a), which equals 0.117 h^−1^ with a standard deviation from the mean of *SD* = 0.028 h^−1^.

For further analysis, days were selected for which the daily average infiltration value (d) was either at its maximum or minimum. The maximum values occurred on 22–23 December (winter), at 0.57 (*SD* = 0.11) and 0.60 (*SD* = 0.09) h^−1^, respectively, while the minimum was recorded on 10 August (summer), at 0.03 (*SD* = 0.02) h^−1^. On 22 and 23 December, a different (weekend) occupancy profile was present in the room compared to the August day, resulting in different levels of moisture gains from occupants.

### 4.2. Short-Term RH Response for Selected Days with High- and Low-Infiltration Levels

The following analysis compares indoor relative humidity responses for the selected winter and summer days, using the material and moisture-load variants defined in Section 2.4.

#### 4.2.1. Painted Gypsum Plasterboard (B) and Painted Clay Board (A)

**Figure 3:**
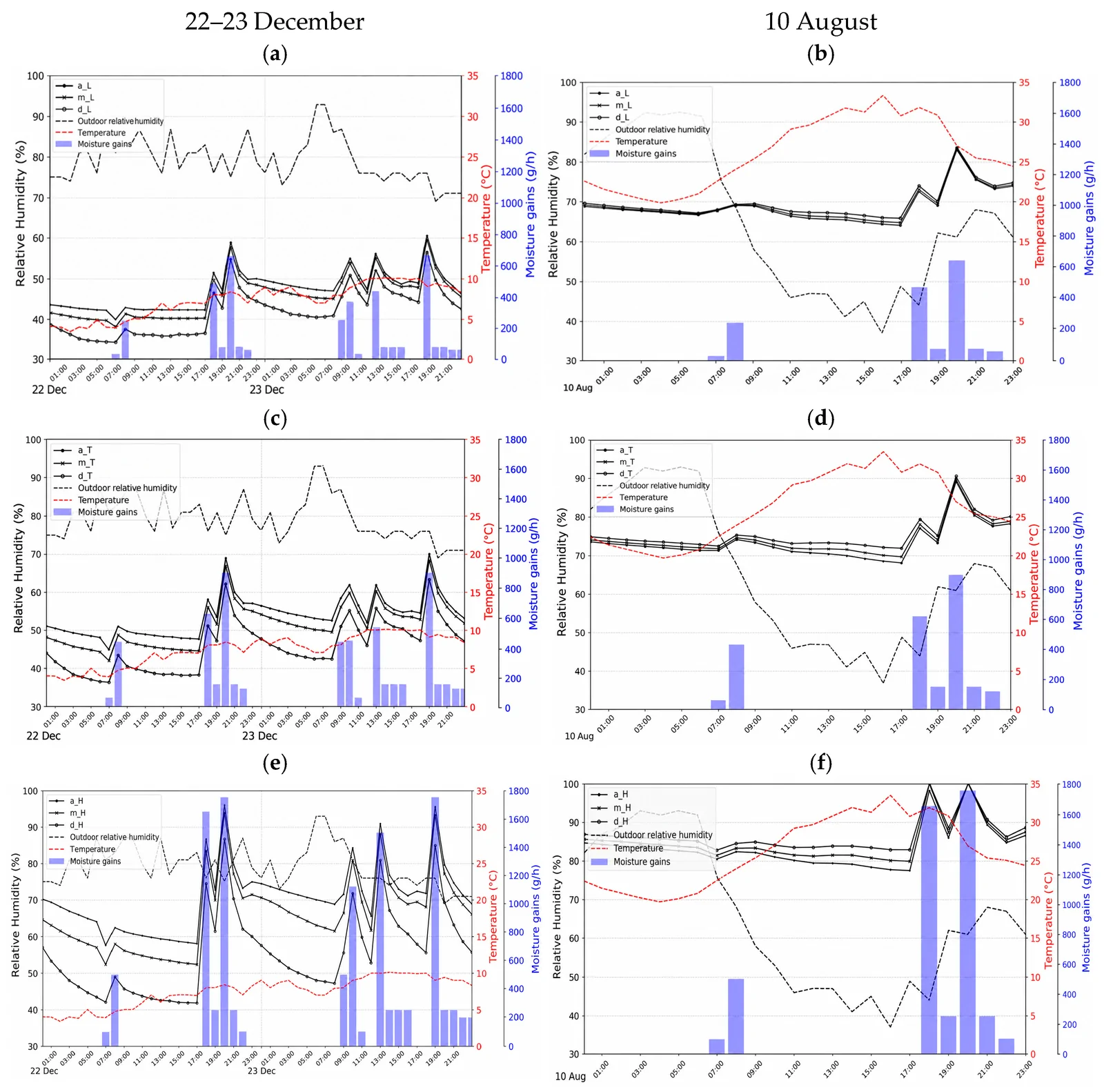
Painted gypsum plasterboard; RH values in the room for 22–23 December and 10 August: (**a**,**b**) low moisture gain levels (L); (**c**,**d**) typical levels (T); (**e**,**f**) high levels (H).

Figure 3 presents the hourly values of various parameters in the interior and surrounding areas of a building with an internal cladding made of painted gypsum plasterboard (B). The three graphs on the left side (a, c, and e) correspond to conditions of high infiltration (22 and 23 December), while the three graphs on the right side (b, d, and f) correspond to the August day with minimal air infiltration. Each horizontal pair of graphs represents increasing levels of moisture gains, starting from low (L) in graphs a and b, through typical (T) in c and d, and ending with very high moisture gains (H) in e and f. The solid black line represents the outdoor relative humidity. The three coloured curves, which follow a similar shape, correspond to the three previously defined simplified methods of accounting for infiltration: a—annual average value, m—monthly average value, and d—daily average value. The dashed black line at the bottom of each graph shows the outdoor temperature. On the secondary axis (right side of each plot), a bar chart indicates the moisture gains.

The differences between the annual, monthly and daily infiltration representations are much more pronounced during the winter high-infiltration period than during the summer low-infiltration day. This behaviour is physically consistent: when infiltration is low, all three averaging approaches converge towards a similar total air exchange rate; when infiltration is high and variable, temporal averaging directly modifies the moisture-removal capacity of the room. Under low and typical moisture gains during the December period, infiltration helps prevent excessive indoor drying while keeping most RH values close to the comfort range. Under high moisture gains, however, even the combined effect of mechanical ventilation and infiltration is insufficient to prevent short-term exceedances above 60% RH. In the low-infiltration August case, indoor RH remains above the comfort range for most or all of the day, and the choice of infiltration representation has only minor influence.

These selected days show that infiltration can either improve or worsen indoor humidity conditions depending on outdoor climate, internal moisture loads and the adopted performance criterion. It should therefore not be interpreted as universally beneficial. In the analysed winter cases, higher infiltration increased moisture removal and reduced RH peaks, but it could also contribute to lower RH under low moisture loads. The fixed mechanical ventilation rate of approximately one air change per hour was not sufficient, by itself, to maintain RH within 40–60% under all moisture-load scenarios.

For the painted clay board (A), the short-term indoor RH response was similar to that obtained for painted gypsum plasterboard (B). This similarity should be interpreted in the context of the identical coating resistance, *s_d_* = 0.5 m, applied to both substrates. The results are consistent with the coating restricting moisture exchange and thereby masking the influence of differences in substrate moisture buffering properties under the analysed conditions. The comparison illustrates the importance of the finished surface for room-scale humidity performance; it does not imply that the underlying boards have equivalent hygric properties.

#### 4.2.2. Unpainted Clay Board (A*)

**Figure 4:**
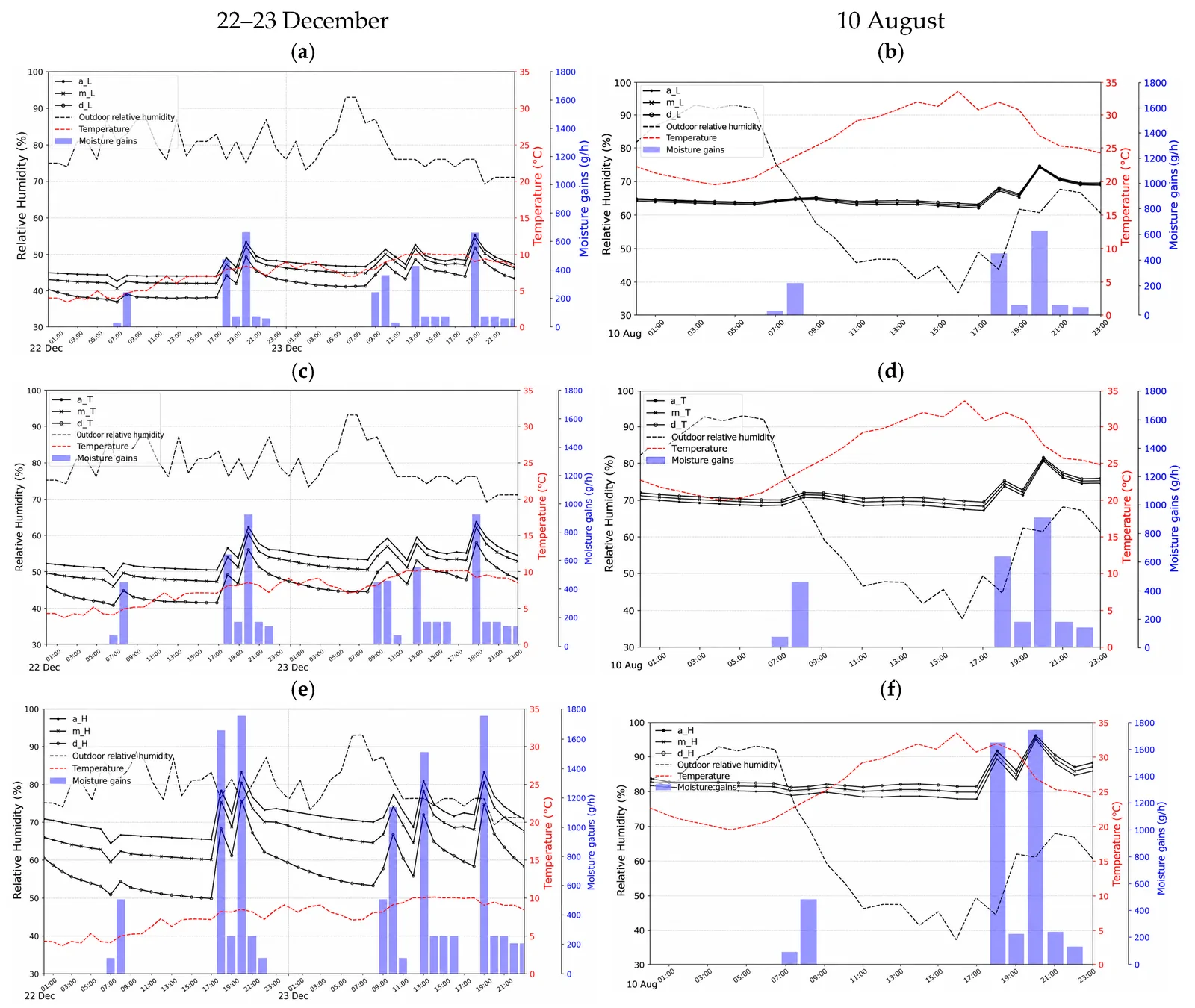
Unpainted clay boards; RH values in the room for 22–23 December and 10 August: (**a**,**b**) low moisture gain levels (L); (**c**,**d**) typical levels (T); (**e**,**f**) high levels (H).

Figure 4 presents the variation in humidity conditions in the simulated room during short periods of highest (December) and lowest (August) air infiltration, in a room with internal cladding made of unpainted clay boards.

The unpainted clay board (A*) provides a stronger moisture buffering response than the two painted variants. Nevertheless, its buffering capacity is not sufficient to maintain RH within 40–60% under all boundary conditions. During the low-infiltration August day, RH remains above the comfort range even for the low moisture-load scenario, and the differences between infiltration representations are negligible. During the high-infiltration December period, the active clay surface reduces the amplitude of short-term RH peaks and produces a more stable response than the painted finishes. However, under high moisture gains, RH still exceeds the comfort range for substantial periods. The results therefore indicate that exposed hygroscopic surfaces can mitigate short-term humidity fluctuations, but their effectiveness remains constrained by the magnitude of moisture emissions and by the available air exchange. 

### 4.3. Whole Year Results

The annual simulations were analysed using the statistical indicators defined in Section 2.5. Therefore, the assessment was extended to cover the entire annual period, introducing a parametric evaluation that reflects the quantitative differences among the various simulation models analysed. Based on year-long simulations with an hourly time step, a series of statistical analyses of the obtained results were conducted, in accordance with the criteria outlined in Section 2.5 and summarised in Table 7. Analysing the values of the basic statistical measures for the analysed material reveals that, regardless of the simplification adopted in modelling air infiltration, (a), (m), or (d), and regardless of the adopted level of moisture gains, (L), (H), or (T), the range of relative humidity (RH) for the material with greater moisture buffering capacity, i.e., the unpainted clay board (A*), is smaller than for materials coated with a vapour-impermeable layer (A and B). 

For variants with low (L) and typical (T) moisture gains, the reduction in the maximum RH range for material (A*) compared to materials (A) and (B) is consistent, averaging 17.6% for variant (L) and 26.0% for variant (T), regardless of the level of simplification in infiltration modelling, whether (a), (m), or (d). In the case of high moisture gains (H), the effect of reducing the RH range is also present, but its magnitude decreases as the level of detail in infiltration modelling increases. Thus, for the annual simplification mode (a), the reduction amounts to 46.5%; for the monthly simplification (m), it is 42.7%; and for the daily simplification (d), it is 37.7.

The median value of relative humidity clearly increases with the intensity of moisture emission, which is an expected observation; however, no distinct relationship with the infiltration model can be identified here. It can be observed that in variants with low moisture gains (L), regardless of the method used to average infiltration, (a), (m), or (d), the median remains at a consistent level of approximately 47–48% across all analysed materials ((A*), (A), and (B)). In contrast, in variants with typical moisture gains (T), the median for material (A*) reaches a marginally higher value, amounting to 53.8% for simplifications (a) and (m), and 53.6% for (d). The differences between the results for individual infiltration simplifications are minimal, not exceeding 0.2 percentage points (p.p.). For high moisture gains (H), material (A*) reaches elevated median values, approximately 69.5%, regardless of the infiltration model used—(a), (m), or (d). For materials with lower sorptivity (A and B), the median RH value increases with the level of detail in the infiltration model—from approximately 66.3% in the annual variant (a) to 67.1% in the daily variant (d) for model A and from 66.6 to 67.3% for model B, though these differences do not exceed 2%. For painted materials, conditions of excessively dry air are observed almost exclusively in the L and T moisture emission variants, with the highest values associated with monthly infiltration modelling. The choice of the monthly model combined with a low level of moisture gains results in a 12% higher median value compared to the annual model. 

The coefficient of variation (*CV*) provides additional information on the dispersion of annual RH values, but it should not be interpreted as a direct comfort indicator. For all analysed variants, *CV* remained below 23%, indicating moderate dispersion of the simulated RH series. However, *CV* decreased with increasing moisture gains because the mean RH increased, even when the absolute RH range became wider. For example, for material (A*) with daily averaged infiltration, *CV* decreased from 20.3% under low moisture gains to 13.3% under high moisture gains, while the RH range increased from 49.7% to 57.1%. Therefore, the range, median and exceedance-based indicators must be interpreted together when assessing the effect of infiltration averaging on indoor humidity performance.

### 4.4. Humidity Comfort

A direct measure of the quality of humidity conditions in an indoor environment is the percentage of hours during which the relative humidity (RH) falls within the accepted comfort range, i.e., 40–60% RH. 

With regard to the number of comfort hours within the 40–60% RH range, the presented results indicate that simplifications in infiltration modelling have a noticeable impact on the estimation of humidity comfort. The extent of this impact is closely dependent on the type of internal finishing materials used and the level of moisture gains within the space. For low moisture gains (L), the proportion of comfort hours remains relatively constant regardless of the infiltration simplification applied. Differences between models (a), (m), and (d) across all materials do not exceed 2.6 percentage points (p.p.). At the same time, material (A*), characterised by high sorption capacity, exhibits the highest proportion of comfort hours (63.6–61.0%), confirming its expected ability to passively stabilise indoor humidity levels. In the case of typical moisture gains (T), the influence of the infiltration model’s level of detail becomes more noticeable. For materials (B) and (A), the differences between the annual (a) and daily (d) approaches amount to 2.7 and 2.9 p.p., respectively. Significant differences are observed, however, under conditions of high moisture gains (H). For material (A*), the proportion of comfort hours drops to as low as 18.0% (annual infiltration model (a)), while the monthly model (m) captures temporary improvements (up to 22.1%). Thus, even material with superior buffering capacity is unable to maintain humidity comfort within the modelled interior. For materials A and B, the comfort hours are slightly higher (approximately 30–31%) and remain stable regardless of the infiltration model adopted, which may be attributed to their limited moisture buffering capacity. 

Table 7 presents, for the analysed variants, an additional evaluation method specifically developed by the authors, which assesses the distribution and duration of simulation results using a percent-hour index. Conditions that go beyond the humidity comfort range in both directions (below and above) are taken into account. The index is calculated as the product of the difference between the current RH value, expressed as a percentage, and the maximum/minimum comfort value and duration of these conditions, expressed in hours. In the case of percent-hours with relative humidity below 40%, a clear influence of the sorption properties of interior surfaces is observed. Material B consistently results in the highest number of hours with excessively dry air. In contrast, material (A*), representing an active clay board without painting, significantly mitigates this issue—particularly under low and typical moisture gain scenarios. For example, under low moisture gains (L), the percent-hour index for RH < 40% for material (B) ranges from 13,009%h with the infiltration model (a) to 14,484%h with model (m), whereas for material (A*) it drops to a range of 9723–11,210%h, representing a reduction of approximately 22% annually compared to material B. Similar differences are observed at typical levels of indoor moisture (T), where for material (A*), the number of percent-hours below the comfort threshold is ca. three times reduced to 1208–1961%h, compared to 3393–4521%h for material (B). For all three materials, the monthly infiltration simulation model shows the highest values of this indicator. At high moisture gains (H), the phenomenon of air dryness virtually disappears for all materials, indicating the dominant influence of moisture emissions on hygrometric conditions in such cases.

Regarding exceedances of the upper threshold of humidity comfort (RH > 60%), the situation is more complex. Under low (L) and typical (T) moisture gains, material (A*) continues to demonstrate favourable properties—effectively reducing the percent-hour index with excessive humidity in comparison to materials (B) and (A). For typical moisture gains (T), material (A*) reaches values in the range of 10,297–11,948%h, whereas material (B) records values between 12,445 and 14,225%h. The highest exceedance values are observed for the daily analysis scheme regardless of the type of sorption material, which clearly highlights the need for short-term infiltration analysis. The observed relation suggests that the highly sorptive material effectively damps short-term increases in humidity, particularly under conditions of moderate use. However, under high moisture gains (H), the opposite effect is observed. Material (A*), despite its buffering properties, exhibits the highest percent-hour index for RH > 60%—ranging from 80,395 to 83,691%h. Again, the highest values of exceedances beyond the comfort zone were obtained for the daily modelling approach. Easy access of water vapour to the highly absorbing material without a vapour barrier coating resulted in ca. 5% deterioration of the conditions in the room. 

### 4.5. Sensitivity to the Vapour Resistance Factor

The influence of the adopted constant vapour resistance factor was evaluated for the unpainted clay board (A*) under high moisture gains (H), using daily-averaged infiltration (d). In this configuration, hourly infiltration rates were averaged separately for each day, and the resulting daily mean was applied as a constant rate throughout that day. The measured dry-cup value, *μ* = 17.4, was compared with constant values of *μ* = 10, 8, and 6. No additional surface coating resistance was imposed, and all other model inputs were unchanged.

Table 8 summarises the results over the common 8760 h evaluation period; annual mean RH ranged from 68.67% to 68.98%, and median RH from 69.49% to 69.82%. Thus, reducing *μ* by approximately 66% relative to the baseline changed these indicators by only 0.31 and 0.33 percentage points, respectively. Annual maximum RH ranged from 94.73% to 96.37%, while annual minimum RH ranged from 39.22% to 41.72%.

Humidity fluctuations showed greater sensitivity. The annual RH range, defined as the annual maximum minus the annual minimum, varied from 53.01 to 57.15 percentage points. The mean daily RH range, calculated as the average of the daily maximum–minimum differences, varied from 13.05 to 16.17 percentage points. The proportion of hours within the adopted comfort interval of 40–60% RH ranged from 16.88% to 19.61%. Nevertheless, RH exceeded 60% during approximately 80–83% of the annual period in all cases.

The small differences in mean and median RH, together with the consistent assessment of predominantly high indoor humidity, support the use of the measured constant *μ* = 17.4 for the investigated A*–H–d configuration. This assessment remained unchanged across the tested *μ* range, even without a paint coating that could restrict moisture exchange with the substrate. The sensitivity analysis also quantifies the influence of μ on RH fluctuations and comfort hours.

## 5. Discussion

The issue of health and comfort of inhabitants in relation to air exchange is widely discussed in the literature. An example is the publication by Mustafa et al. [4], in which the authors conducted a frequently cited review on the impact of ventilation on occupant health and comfort. However, their analysis primarily addressed ventilation from the perspective of air quality and did not explicitly examine the role of air infiltration, enclosure tightness, or even indoor relative humidity. San Jose and Perez-Camanyo [29] presented a more specific study on the impact of highly variable infiltration on indoor air quality. This article also fails to mention air humidity aspects. This suggests that, although ventilation and infiltration have been analysed in terms of general air quality, the impact of different levels of simplification in infiltration modelling on indoor humidity dynamics and the performance of moisture-buffering materials remains practically unaddressed in the literature. That is why the authors decided to analyse the impact of infiltration variability on the relative air humidity in the building in such detail.

In simulation analyses using the WUFI Plus software, researchers most commonly assume an average annual air infiltration rate ranging from 0.1 to 0.5 h^−1^ [6,7,8,9,10,11], claiming that such a simplification does not significantly distort the moisture dynamics between the material and the indoor environment. However, the presented analyses indicated that a more accurate representation of indoor humidity conditions can be achieved by adopting a more precise air infiltration model. This is particularly important when analysing the behaviour of moisture-buffering materials in conjunction with a mechanical supply and exhaust ventilation system. 

Accurate representation of the dynamics of infiltration air exchange is crucial for assessing humidity comfort in buildings, particularly in spaces where materials with limited moisture buffering capacity are used or where intensive occupancy leads to high moisture loads. The level of detail in modelling air infiltration (annual, monthly, or daily) has a noticeable impact on the results, although it is less significant than the influence of the material properties themselves.

The simulation tests we carried out were for three levels of infiltration simplification (annual, monthly, or daily), three interior surface finishing materials (A, A*, or B) and three scenarios of humidity gains in the room (L, T, or H). The obtained results confirmed a consistent decreasing trend between the value of MBV and the RH range: the more hygroscopic the material and the greater its capacity for moisture buffering (i.e., the higher the MBV), the lower the RH data range. A similar relationship was observed by Larcher et al. [54], who, in their research, analysed the relationship between RH values obtained from building simulations and the MBV of various types of plasters. The interaction between ventilation rate and material moisture buffering capacity has also been highlighted by Woloszyn et al. [56], who showed that the humidity-regulating effect of hygroscopic materials strongly depends on the air exchange strategy adopted in the model.

The conducted research analysis showed that a material with high MBV, such as clay board (A*), demonstrates greater activity in buffering moisture under conditions of high moisture load (H), as also confirmed by the findings of Rode et al. [20] and Antretter et al. [57]. The observed effect was particularly visible in scenarios with dynamically varying internal moisture gains. In such cases, the accuracy of the numerical infiltration model becomes particularly important, as evidenced by the example of the daily variant (d) and the analysed material (A*). However, the differences between modelling variants were most pronounced under high moisture-load conditions. According to the obtained results, in these conditions, the adopted moisture buffering capacity of the simulated building envelope and the overall air exchange rate were too low to protect the interior from excessive relative humidity (RH). This conclusion refers specifically to the analysed configuration and adopted ventilation assumptions. Within the range of low moisture gains and low internal humidity, moisture buffering was also an effective measure against air dryness. This finding does not align with the observations of Osanyintola and Simonson [58], which suggest that moisture buffering by hygroscopic materials becomes significant only after a certain indoor RH threshold is exceeded. The discrepancy may result from differences in initial humidity levels and material sorption characteristics.

A more detailed statistical analysis supports the interpretation presented above. The comparison of the three measures (range, median, and coefficient of variation) reveals significant interrelations: in cases of higher median values, even with a relatively wide range, the coefficient of variation is considerably lower. It should be noted that the coefficient of variation is mathematically influenced by the magnitude of the mean value. This indicates that the distribution of RH values is more concentrated and that hygrothermal conditions are more stable, despite the broad spread of extreme values. This effect is particularly pronounced for material (A*), confirming its superior capacity for passive moisture regulation.

In addition, the simplifications adopted in modelling air exchange do not significantly distort the moisture properties of the given material-space system. This observation applies to scenarios with low and medium moisture input. Under limited moisture input, a simplified approach (e.g., the monthly or even annual model) may be justified without substantially affecting the comfort rating. Such simplification should not be extrapolated to cases with high internal moisture gains. This finding contributes practical guidance for practitioners who must balance modelling complexity against project constraints, addressing concerns raised by Clarke [59] regarding the appropriate level of detail in building simulation.

The sensitivity analysis reported in Table 8 provides quantitative support for adopting the measured constant vapour resistance factor. For the unpainted clay board under high moisture gains and daily-averaged infiltration (A*–H–d), substantial variation in *μ* produced small differences in annual mean and median RH, while the assessment of predominantly high indoor humidity remained consistent. The absence of a paint coating ensured that this comparison was not constrained by an additional coating resistance. The results therefore support the adopted simplification for the investigated configuration, while quantifying its influence on RH fluctuations and comfort hours.

A low level of comfort, despite the material’s high hygroscopicity, indicates the saturation of its sorption capacity and highlights the necessity of supporting moisture control through mechanical systems. This result indicates a risk of moisture accumulation in hygroscopic materials in the absence of adequate dehumidification, which, over time, may lead to saturation of the material and its impact on maintaining excessively high indoor air humidity [60]. Whole-building simulations performed by Woloszyn et al. [56] similarly indicated that, under insufficient air exchange, moisture-buffering materials may experience temporary saturation, reducing their ability to limit elevated RH levels.

From a methodological perspective, the proposed indicators, calculated on the basis of hourly simulation outputs, allow for a more pronounced analysis of humidity conditions beyond the comfort range and extended evaluation of the effects of the material and installation solutions used. The purpose of this evaluation is to illustrate not only the values exceeding the comfort range but also to account for the duration of these conditions. This approach may be useful in future studies aimed at assessing the practical implications of different modelling assumptions or material choices. It responds to the need identified by Fanger [61] and Toftum et al. [62] for more comprehensive methods of evaluating indoor comfort conditions. 

In summary, the sorption properties of internal finishing materials play a crucial role in shaping humidity comfort in a building. Actively moisture-buffering materials, such as unpainted clay boards, can significantly reduce the risk of air drying under low to moderate moisture gains. However, under conditions of high moisture emissions, their effectiveness diminishes, and they may even contribute to maintaining excessively high indoor humidity if adequate dehumidification conditions are not provided. Nevertheless, a detailed assessment of these conditions is only possible with an appropriately selected air infiltration modelling approach, which takes into account its immense variability and allows especially to detect threats related to high humidity levels.

The quantitative results obtained in this study are inherently linked to the specific material solutions adopted and the prevailing climatic conditions; consequently, they cannot be directly extrapolated to vastly different climates. This opens up a significant avenue for future research into material solutions in other climatic zones, potentially involving also different criteria for assessing the indoor moisture environment. Humid climates, in particular, present a distinct challenge: the humidity comfort range typically adopted in Europe (40–60%) may prove impossible to maintain—even partially—given the high external moisture loads characteristic of such regions. Nevertheless, the authors believe this study conveys a universal message regarding the significant impact of air infiltration and, by extension, the importance of how it is accounted for in simulation models. Specifically, under conditions of high moisture load, relying on long-term average values for this additional air exchange leads to an inaccurate assessment of actual conditions. Therefore, the impact of infiltration variability demonstrated in this article warrants detailed analysis, particularly in humid climates.

## 6. Conclusions

Based on the conducted analyses, the following conclusions can be drawn:

- Temporal averaging of infiltration affects the predicted indoor humidity response, but the magnitude of this effect depends strongly on moisture-load intensity and material hygroscopicity. Under low moisture gains, differences in comfort hours between annual, monthly and daily infiltration representations did not exceed 2.6 percentage points, indicating that annual averaging may be sufficient for preliminary assessment.
- Under high moisture gains—reaching a peak emission of approximately 1.8 kg/h—the adopted infiltration resolution becomes important. Daily infiltration values provide a more representative description of short-term moisture removal and should be used when the analysis focuses on RH peaks, exceedance duration or the effectiveness of moisture buffering finishes.
- The unpainted clay board (A*) improved humidity stabilisation compared with painted materials, but it did not maintain RH within the 40–60% comfort range under all boundary conditions. Passive moisture buffering therefore reduces short-term RH fluctuations but cannot compensate for persistently high internal moisture emissions or insufficient moisture removal.
- Surface finishing substantially influenced the moisture buffering performance of the analysed materials. The painted clay board (A) and painted gypsum plasterboard (B), modelled with the same coating resistance of *s_d_* = 0.5 m, produced similar indoor RH responses. This convergence indicates that the contribution of substrate differences was reduced under the adopted surface resistance and boundary conditions. The finding is conditional on these assumptions and should not be generalised to other coating systems. Simulations and design assessments should therefore account for the vapour resistance of surface finishes alongside the hygric properties of the substrate.
- The use of exceedance-based indicators provides a more informative assessment than annual mean RH alone. Percent-hour indicators make it possible to distinguish between short, mild deviations and longer or more severe periods outside the 40–60% RH range.
- For energy-efficient and mechanically ventilated buildings, the choice of infiltration time-averaging should be linked to the purpose of the simulation. Annual or monthly averages may be adequate for general annual screening, whereas daily values are recommended for detailed humidity-comfort assessment, high moisture-load scenarios and evaluation of active moisture-buffering materials.
- The results demonstrate that oversimplified infiltration assumptions may lead to misleading interpretation of indoor humidity stability. The proposed approach can support more consistent hygrothermal modelling of energy-efficient rooms by matching the infiltration input resolution to the required level of design accuracy.

## Figures and Tables

**Table 1:** Thermal characteristics of the building components for variant (B).

Building Component	Design (Outwards)	U W/(m^2^⋅K)
Exterior wall (South and West)	12.5 mm cement–lime plaster 120 mm expanded polystyrene (EPS) 250 mm solid brick 12.5 mm gypsum plasterboard	0.20
Roof (Horizontal)	1 mm bituminous paper 200 mm mineral wool 1 mm vapour barrier (s_d_ = 1500 m) 10 mm concrete screed, top layer 20 mm concrete screed, mid layer 10 mm concrete screed, bottom layer 200 mm concrete, w/c = 0.5 12.5 mm gypsum plasterboard	0.18
Interior wall	12.5 mm gypsum plasterboard 120 mm solid brick 12.5 mm gypsum plasterboard	1.65
Floor	20 mm laminate flooring 50 mm concrete screed, top layer 80 mm mineral wool 240 mm concrete, w/c = 0.5 15 mm cement–lime plaster	0.38
Window		0.90

**Table 2:** Unit moisture emission, in grams per unit.

	Unit	Moisture Emission		
		Low (L)	Typical (T)	High (H)
Preparing breakfast	g/meal	170	280	250
Preparing lunch	g/meal	250	320	950
Preparing dinner	g/meal	580	750	1750
People	g/(h⋅person)	30	60	100
Dishwasher operation	g/day	50	150	450
Cleaning	g/m^2^	5	5	15
Plants	g/h	2.5	2.5	2.5

**Table 3:** Daily moisture-load scenarios for the analysed room.

Time Interval	Monday–Friday				Saturday–Sunday			
	Occupancy Schedule	Moisture Gains, g/h			Occupancy Schedule	Moisture Gains, g/h		
		**L**	**T**	**H**		**L**	**T**	**H**
0:00–6:00		2.5	2.5	2.5		2.5	2.5	2.5
6:00–7:00	1 adult	32.5	62.5	102.5		2.5	2.5	2.5
7:00–8:00	2 adults, 1 child	247.5	432.5	502.5		2.5	2.5	2.5
8:00–9:00		2.5	2.5	2.5	2 adults, 1 child	247.5	432.5	502.5
9:00–10:00		2.5	2.5	2.5	2 adults, 1 child	367.5	442.5	1122.5
10:00–11:00		2.5	2.5	2.5	1 adult	32.5	62.5	102.5
11:00–12:00		2.5	2.5	2.5		2.5	2.5	2.5
12:00–13:00		2.5	2.5	2.5	1 adult	432.5	532.5	1502.5
13:00–16:00		2.5	2.5	2.5		77.5	152.5	252.5
16:00–17:00		2.5	2.5	2.5		2.5	2.5	2.5
17:00–18:00	2 adults, 1 child	477.5	622.5	1652.5		2.5	2.5	2.5
18:00–19:00	2 adults, 1 child	77.5	152.5	252.5	2 adults, 1 child	657.5	902.5	1752.5
19:00–20:00	2 adults, 1 child	657.5	902.5	1752.5	2 adults, 1 child	77.5	152.5	252.5
20:00–21:00	2 adults, 1 child	77.5	152.5	252.5	2 adults, 1 child	77.5	152.5	252.5
21:00–22:00	1 adult	62.5	122.5	102.5	2 adults	62.5	122.5	202.5
22:00–23:00		2.5	2.5	2.5	2 adults	62.5	122.5	202.5
23:00–0:00		2.5	2.5	2.5		2.5	2.5	2.5

**Table 4:** The moisture content (*w*) of materials as a function of relative humidity (RH) for clay board (A*) and gypsum plasterboard (B).

RH, %	20	30	40	50	60	65	70	75	80	85	90	93	95
Clay board w, kg/m^3^	14	14.3	15.4	16.6	17.8	-	19.7	21.1	23.1	25.7	30.1	-	36.8
Gypsum plasterboard w, kg/m^3^	1.5	-	-	3.6	-	5.2	-	-	6.3	-	11	17	19

**Table 5:** Values from measurements and calculations for clay board (A*).

	μ_dry_	λ	ϕ	$\xi_{w}^{33-75\%}$	d_p_	MBV_ideal_	MBV_practical_
	**-**	**W/(m** **⋅** **K)**	**-**	**kg/m^3^**	**cm**	**g/(m^2^** **⋅** **%RH)**	**g/(m^2^** **⋅** **%RH)**
Measured	17.4	0.19	–	–	–	–	1.88
Calculated	–	–	0.39	30.0	2.5	1.63	–

**Table 6:** Basic properties from WUFI Plus database.

Material Name	ρ kg/m^3^	ϕ -	λ W/(m·K)	c_p_ J/(kg·K)	μ -
gypsum plasterboard	850	0.65	0.20	850	8.3
clay board	1500	–	–	950	–

**Table 7:** Statistical measures of humidity comfort for materials.

	Moisture Gain	Painted Gypsum Plasterboard (B)			Painted Clay Board (A)			Unpainted Clay Board (A*)		
		Simulation Model			Simulation Model			Simulation Model		
		Annual (a)	Monthly (m)	Daily (d)	Annual (a)	Monthly (m)	Daily (d)	Annual (a)	Monthly (m)	Daily (d)
Comfort hours [%]	low L	60.2	58.3	59.1	60.8	58.9	59.8	63.6	61.0	62.0
	typical T	63.2	59.8	60.5	64.6	61.0	61.7	66.5	63.4	64.5
	high H	30.4	31.3	30.2	30.1	31.4	30.4	18.0	22.1	19.6
Max. Difference [%]	low L	59.5	61.0	60.5	59.2	60.6	60.1	48.6	50.4	49.7
	typical T	61.4	63.8	63.4	60.9	63.2	62.9	48.1	50.3	50.6
	high H	63.7	65.7	67.9	63.3	65.4	67.4	49.6	53.0	57.1
Coefficient of variation [%]	low L	21.6	22.4	22.1	21.3	22.1	21.8	19.7	20.6	20.3
	typical T	19.0	20.1	19.8	18.7	19.8	19.5	16.7	17.8	17.5
	high H	17.2	18.3	18.2	16.9	18.0	17.9	12.0	13.3	13.3
Median [%]	low L	47.3	47.5	47.5	47.2	47.3	47.3	47.9	47.9	47.7
	typical T	53.0	53.1	53.1	52.8	53.0	52.9	53.8	53.8	53.6
	high H	66.6	66.8	67.3	66.3	66.6	67.1	69.4	69.5	69.5
Percent-hours below 40% [%h]	low L	13,009	14,484	13,422	12,758	14,254	13,191	9723	11,210	10,188
	typical T	3393	4521	3942	3191	4300	3745	1208	1961	1751
	high H	7	66	109	6	51	94	0	0	1
Percent-hours above 60% [%h]	low L	4151	4414	4629	3793	4046	4238	2452	2733	2835
	typical T	12,445	13,416	14,225	11,626	12,560	13,304	10,297	11,516	11,948
	high H	76,660	78,535	81,263	74,915	76,770	79,524	80,395	81,413	83,691

**Table 8:** Sensitivity of indoor relative humidity to the vapour resistance factor of the unpainted clay board (A*) under high moisture gains (H) and daily-averaged infiltration (d).

μ [–]	Mean RH [%]	Median RH [%]	Minimum RH [%]	Maximum RH [%]	Annual RH Range [p.p.]	Mean Daily RH Range [p.p.]	Hours Within 40–60% RH [%]	Time Above 60% RH [h]
6	68.98	69.82	41.72	94.73	53.01	13.05	16.88	7281
8	68.88	69.73	41.17	95.35	54.18	13.93	17.58	7220
10	68.81	69.65	40.66	95.69	55.03	14.60	18.21	7165
17.4 (baseline)	68.67	69.49	39.22	96.37	57.15	16.17	19.61	7039

## Data Availability

The original contributions presented in this study are included in the article. Further inquiries can be directed to the corresponding author.

## Abbreviations

a	annual
A	painted prefabricated clay board
A*	unpainted prefabricated clay board
B	painted gypsum plasterboard (base variant)
d	daily, specimen thickness, m
H	high moisture gains
ITB	Building Research Institute
L	low moisture gains
m	monthly
MBC	moisture buffering capacity
MBV	moisture buffering value
NZEB	Nearly Zero Energy Building
RH	relative humidity
S-Ex	supply–exhaust ventilation
T	typical moisture gains
Physical quantities	
b_m_	moisture effusivity, kg/(m^2^⋅Pa⋅s^1/2^)
c_p_	specific heat capacity, J/(kg·K)
CV	coefficient of variation, %
D_w_	moisture diffusivity of the material, m^2^/s
d_p_	moisture penetration depth, cm
G	water vapour flow rate, kg/s
m	mass, g
Δp	water vapour pressure difference, Pa
p_sat_	water vapour saturation pressure at 23 °C, Pa
Ph	percentage hours, %h
S	exposed surface area, m^2^
s_d_	equivalent air layer thickness, m
SD	standard deviation, %
t_p_	time period, s
U	thermal transmittance, W/(m^2^⋅K)
U(RH)	expanded uncertainty of relative humidity, % RH
U(w)	expanded uncertainty of moisture content, kg/m^3^
w	moisture content mass by volume, kg/m^3^
Greek symbols	
δ_a_	vapour permeability of air, kg/(m·s·Pa)
δ_p_	water vapour permeability, kg/(m⋅s⋅Pa)
ξ_w_	moisture capacity, kg/m^3^
µ	water vapour resistance factor, -
X	arithmetic mean, %
ϕ	porosity, -
λ	thermal conductivity, W/(m·K)
ρ	density, kg/m^3^

## References

[1] Zu K., Qin M., Rode C., Libralato M. (2020). Development of a moisture buffer value model (MBM) for indoor moisture prediction. Appl. Therm. Eng..

[2] Woods J., Winkler J., Christensen D. (2013). Moisture Penetration Depth Model for Estimating Moisture Buffering in Buildings.

[3] Simonson C.J., Salonvaara M., Ojanen T. (2001). Improving Indoor Climate and Comfort with Wooden Structures.

[4] Mustafa M., Cook M.J., McLeod R.S. (2025). A critical review of ventilation effectiveness in naturally ventilated spaces form the perspective if sustainability and health. Build. Environ..

[5] Yoshino H., Mitamura T., Hasegawa K. (2009). Moisture buffering and effect of ventilation rate and volume rate of hygrothermal materials in a single room under steady state exterior conditions. Build. Environ..

[6] Rode C., Grau K. (2008). Moisture buffering and its consequence in whole building hygrothermal modeling. J. Build. Phys..

[7] Li Y., Fazio P., Rao J. (2012). An investigation of moisture buffering performance of wood paneling at room level and its buffering effect on a test room. Build. Environ..

[8] Fang J., Lai Q., Zhang H., Ren P., Tang M., Li X., Feng C. (2024). Influence of materials and surface parameters on the moisture buffering in office buildings: A sensitivity analysis of China. Case Stud. Therm. Eng..

[9] Kupczak A., Sadłowska-Sałęga A., Krzemień L., Sobczyk J., Radoń J., Kozłowski R. (2018). Impact of paper and wooden collections on humidity stability and energy consumption in museums and libraries. Energy Build..

[10] Xia D., Zhong Z., Huang Y., Zou Y., Lou S., Zhann Q., Gua J., Yang J., Gua T. (2023). Impact of coupled heat and moisture transfer on indoor comfort and energy demand for residential buildings in hot-humid regions. Energy Build..

[11] Yang X., Ge H., Fazio P., Rao J. (2014). Evaluation of parameters influencing the moisture buffering potential of hygroscopic materials with BSim simulations. Buildings.

[12] Straube J.F., deGraauw J.P. (2001). Indoor air quality and hygroscopically active materials. ASHRAE Trans..

[13] Ding D., Rasmussen O.S., Qin M. (2024). Moisture buffer value for hygroscopic materials with different thicknesses. Build. Environ..

[14] Albelda-Estellés Ness M.C. Indoor relative humidity: Relevance for health, comfort, and choice of ventilation system. Proceedings of the 3rd Valencia International Biennial of Research in Architecture.

[15] Fang L., Clausen G., Fanger P.O. (1998). Impact of temperature and humidity on perception of indoor air quality during immediate and longer whole-body exposures. Indoor Air.

[16] Liu P., Justo Alonso M., Mathisen H.M., Halfvardsson A., Simonson C. (2023). Understanding the role of moisture recovery in indoor humidity: An analytical study for a Norwegian single-family house during heating season. Build. Environ..

[17] TenWolde A., Walker I.S. (2001). Interior moisture design loads for residences. Proceedings of Performance of Exterior Envelopes of Whole Buildings VIII: Integration of Building Envelopes, Atlanta, GA, USA, 2–7 December 2001.

[18] Eric M., Tanvir K., Bereket A.N., Charles R.W. (2024). Estimating internal moisture generation rates in recently constructed, occupied homes in the southeastern United States. Sci. Technol. Built Environ..

[19] Smith K.M., Svendsen S. (2016). The effect of a rotary heat exchanger in room-based ventilation on indoor humidity in existing apartments in temperate climates. Energy Build..

[20] Rode C., Peuhkuri R.H., Mortensen L.H., Hansen K.K., Time B., Gustavsen A., Ojanen T., Ahonen J., Svennberg K., Arfvidsson J. (2005). Moisture Buffering of Building Materials.

[21] Simonson C.J., Salonvaara M., Ojanen T. (2004). Heat and mass transfer between indoor air and a permeable and hygroscopic building envelope: Part I—Field measurements. J. Build. Phys..

[22] Winkler M., Nore K., Antretter F. (2014). Impact of the Moisture Buffering Effect of Wooden Materials on Energy Demand and Comfort Conditions. https://publica.fraunhofer.de/handle/publica/386435.

[23] Ferreira C., de Freitas V.P., Delgado J.M.P.Q. (2019). The influence of hygroscopic materials on the fluctuation of relative humidity in museums located in historical buildings. Stud. Conserv..

[24] Liuzzi S., Stefanizzi P. (2016). Experimental study on hygrothermal performances of indoor covering materials. Int. J. Heat Technol..

[25] Shang Y., Tariku F. (2021). Hempcrete building performance in mild and cold climates: Integrated analysis of carbon footprint, energy, and indoor thermal and moisture buffering. Build. Environ..

[26] McGregor F., Heath A., Fodde E., Shea A. (2014). Conditions affecting the moisture buffering measurement performed on compressed earth blocks. Build. Environ..

[27] Cascione V., Maskell D., Shea A., Walker P. (2019). A review of moisture buffering capacity: From laboratory testing to full-scale measurement. Constr. Build. Mater..

[28] Ranesi A., Veiga R., Faria P. (2024). The different effect of two paint systems on moisture buffer capacity of traditional and modern plasters. J. Build. Eng..

[29] San Jose R., Perez-Camanyo J.L. (2023). Modelling infiltration rate impacts on indoor air quality. Int. J. Thermofluids.

[30] Shi C., Zhang H., Rui J., Chen Y., Xuan Y., Zhang W., Wang Z., Zhu Y., Wang F., Wang P., Shen C., Liu J. (2019). An experimental investigation of moisture buffering value for composite hygroscopic materials under different air-flow conditions. Proceedings of the 11th International Symposium on Heating, Ventilation and Air Conditioning (ISHVAC 2019).

[31] Ryan B., Bristow D.N. (2024). Investigating the influence of material properties, infiltration, and climatic conditions on hygrothermal risk of building envelopes. Int. J. Technol..

[32] (2025). DesignBuilder Software. https://designbuilder.co.uk.

[33] Fraunhofer Institute for Building Physics WUFI^®^ Plus—Building Simulation Software. https://wufi.de.

[34] (2016). Hygrothermal Performance of Building Materials and Products—Determination of Water Vapour Transmission Properties—Cup Method.

[35] (2022). Hygrothermal Performance of Building Materials and Products—Determination of Hygroscopic Sorption Properties.

[36] (1998). Thermal Insulation—Determination of Steady-State Thermal Resistance and Related Properties—Heat Flow Meter Apparatus.

[37] (2002). Thermal Performance of Building Materials and Products—Determination of Thermal Resistance by Means of Guarded Hot Plate and Heat Flow Meter Methods—Products of High and Medium Thermal Resistance.

[38] (2017). Standard Test Method for Steady-State Thermal Transmission Properties by Means of the Heat Flow Meter Apparatus.

[39] Rode C., Peuhkuri R., Time B., Svennberg K., Ojanen T., Mukhopadhyaya P., Kumaran M., Dean S.W. (2007). Moisture buffer value of building materials. J. ASTM Int..

[40] Latif E., Lawrence M., Shea A., Walker P. (2015). Moisture buffer potential of experimental wall assemblies incorporating formulated hemp-lime. Build. Environ..

[41] Abadie M.O., Mendonça K.C. (2009). Moisture performance of building materials: From material characterization to building simulation using the moisture buffer value concept. Build. Environ..

[42] Claytec Clayboard Heavy LEMIX D22/D16. https://claytec.de/en/product/claydry-building/clayboard-heavy-lemix-d22-d16/.

[43] Goto Y., Wakili K.G., Frank T., Stahl T., Ostermeyer Y., Ando N., Wallbaum H. (2012). Heat and moisture balance simulation of a building with vapor-open envelope system for subtropical regions. Build. Simul..

[44] Hall C., Lo G.J., Hamilton A. (2024). Water vapour permeability of inorganic construction materials. Mater. Struct..

[45] Rode C., Hens H., Janssen H. (2008). IEA Annex 41 Whole Building Heat, Air, Moisture Response: Closing Seminar. Proceedings of the NordicBuildingPhysics Conference, Copenhagen, Denmark, 19 June 2008.

[46] Antretter F., Sauer F., Schöpfer T., Holm A. Validation of a hygrothermal whole building simulation software. In Proceedings of Building Simulation 2011: 12th Conference of International Building Performance Simulation Association.

[47] Ministry of Development and Technology (2022). Announcement of the Minister of Development and Technology of 15 April 2022 on the Publication of the Consolidated Text of the Regulation of the Minister of Infrastructure on the Technical Conditions to be Met by Buildings and Their Location (Journal of Laws 2022, item 1225).

[48] Climat.OneBuilding.Org. Free Weather Data. https://climate.onebuilding.org/.

[49] Künzel H.M. (1995). Simultaneous Heat and Moisture Transport in Building Components: One- and Two-Dimensional Calculation Using Simple Parameters.

[50] Abuku M., Janssen H., Roels S. (2009). Impact of wind-driven rain on historic brick wall buildings in a moderately cold and humid climate: Numerical analyses of mould growth risk, indoor climate and energy consumption. Energy Build..

[51] Hansen T.K., Bjarløv S.P., Peuhkuri R.H., Hansen K.K. (2019). The effects of wind-driven rain on the hygrothermal conditions behind wooden beam ends and at the interfaces between internal insulation and existing solid masonry. Energy Build..

[52] Hansen T.K., Bjarløv S.P., Peuhkuri R.H., Hansen K.K. (2018). Performance of hydrophobized historic solid masonry—Experimental approach. Constr. Build. Mater..

[53] Amorim M., de Freitas V.P., Torres I., Kisilewicz T., Berardi U. (2020). The influence of moisture on the energy performance of retrofitted walls. MATEC Web Conf..

[54] Larcher M., Leonardi E., Troi A., Stefani A., Nerobutto G., Herrera-Avellanosa D. (2025). Assessing the impact of moisture buffering properties of materials on indoor environmental quality: A study on a recycled material plaster. Build. Environ..

[55] Wang M., Lu Z. (2022). Study on the hygroscopic performances of poplar wood fiber biomass brick. Int. Commun. Heat Mass Transf..

[56] Woloszyn M., Kalamees T., Abadie M.O., Steeman M., SasicKalagasidis A. (2009). The effect of combining a relative humidity sensitive ventilation system with the moisture-buffering capacity of materials on indoor climate and energy efficiency of buildings. Build. Environ..

[57] Antretter F., Mitterer C., Jung S.M. Use of moisture-buffering tiles for indoor climate stability under different climatic requirements. Proceedings of the 7th International Conference on Indoor Air Quality, Ventilation & Energy Conservation in Buildings (IAQVEC 2010).

[58] Osanyintola O.F., Simonson C.J. (2006). Moisture buffering capacity of hygroscopic building materials: Experimental facilities and energy impact. Energy Build..

[59] Clarke J.A. (2001). Energy Simulation in Building Design.

[60] Kaczorek D., Basińska M. (2024). Effect of using moisture-buffering finishing materials and DCV systems on environmental comfort and energy consumption in buildings. Energies.

[61] Fanger P.O. (1970). Thermal Comfort.

[62] Toftum J., Jørgensen A.S., Fanger P.O. (1998). Upper limits for indoor air humidity to avoid uncomfortably humid skin. Energy Build..

